# Burden of micronutrient deficiencies by socio-economic strata in children aged 6 months to 5 years in the Philippines

**DOI:** 10.1186/1471-2458-13-1167

**Published:** 2013-12-11

**Authors:** Simon Wieser, Rafael Plessow, Klaus Eichler, Olivia Malek, Mario V Capanzana, Imelda Agdeppa, Urs Bruegger

**Affiliations:** 1 Winterthur Institute of Health Economics, Zurich University of Applied Sciences, Gertrudstrasse 15, CH-8401 Winterthur, Switzerland; 2 Department of Science and Technology, Food and Nutrition Research Institute, Metro Manila, Philippines

**Keywords:** Micronutrient deficiencies, Iron, Vitamin A, Zinc, Cost-of-illness, Burden of disease, Health economics, Philippines, DALY, Production losses, Intangible costs

## Abstract

**Background:**

Micronutrient deficiencies (MNDs) are a chronic lack of vitamins and minerals and constitute a huge public health problem. MNDs have severe health consequences and are particularly harmful during early childhood due to their impact on the physical and cognitive development. We estimate the costs of illness due to iron deficiency (IDA), vitamin A deficiency (VAD) and zinc deficiency (ZnD) in 2 age groups (6–23 and 24–59 months) of Filipino children by socio-economic strata in 2008.

**Methods:**

We build a health economic model simulating the consequences of MNDs in childhood over the entire lifetime. The model is based on a health survey and a nutrition survey carried out in 2008. The sample populations are first structured into 10 socio-economic strata (SES) and 2 age groups. Health consequences of MNDs are modelled based on information extracted from literature. Direct medical costs, production losses and intangible costs are computed and long term costs are discounted to present value.

**Results:**

Total lifetime costs of IDA, VAD and ZnD amounted to direct medical costs of 30 million dollars, production losses of 618 million dollars and intangible costs of 122,138 disability adjusted life years (DALYs). These costs can be interpreted as the lifetime costs of a 1-year cohort affected by MNDs between the age of 6–59 months. Direct medical costs are dominated by costs due to ZnD (89% of total), production losses by losses in future lifetime (90% of total) and intangible costs by premature death (47% of total DALY losses) and losses in future lifetime (43%). Costs of MNDs differ considerably between SES as costs in the poorest third of the households are 5 times higher than in the wealthiest third.

**Conclusions:**

MNDs lead to substantial costs in 6-59-month-old children in the Philippines. Costs are highly concentrated in the lower SES and in children 6–23 months old. These results may have important implications for the design, evaluation and choice of the most effective and cost-effective policies aimed at the reduction of MNDs.

## Background

Micronutrient deficiencies (MNDs) are a chronic lack of vitamins and minerals and constitute a huge public health problem. According to WHO, worldwide nearly one in two preschool children suffer from anemia, many of them because of iron deficiency [1,2], and nearly 100 million preschool children suffer from VAD [3]. Severe and widespread deficiencies also prevail for iodine, zinc and a number of other micronutrients [4]. MNDs are sometimes called hidden hunger, as they are not as visible as malnutrition due to insufficient calorie and protein intake. They have severe health consequences and are particularly harmful during pregnancy and early childhood due to their impact on the physical and cognitive development of children [5,6].

Previous studies have shown that the impact of MNDs on public health can be substantial for countries with high prevalence rates [7-11]. Studies evaluating the impact of a disease are called “cost-of-illness studies” when they focus on its economic costs in terms of medical costs and productivity losses and “burden-of-disease studies” when they focus on its human costs in terms of years lived with disability and years of life lost. These studies are often carried out in order to draw the attention of policy makers to the impact of specific diseases and can thus be of utmost importance for public health priority setting. For MND this is specifically relevant, as effective interventions exist [12-15]. UN development agencies and NGOs have rated interventions focussing on MNDs as a top development priority and these interventions are part of the efforts to reach the United Nations Millennium Development Goals [16,17].

This study is part of a more extensive project on the cost-effectiveness of food fortification for 6-59-month-old children. In a first step we examined whether food fortification is effective in controlled trials [12]. In a second step, which is the subject of the present study, we develop a model of cost consequences of MNDs. In a third step we will evaluate the cost-effectiveness of food fortification interventions in the Philippines.

We chose the Philippines for our cost-of-illness study because it is a large country in which MNDs are still considered to be a public health issue. Furthermore, detailed data on the nutritional status of the population is available.

In this study we estimate the economic and human costs of illness due to iron, vitamin A and zinc deficiencies in 6-59-month-old Filipino children by socio-economic strata with a model simulating the lifetime consequences of MNDs.

## Methods

### Ethics statement

The datasets used in this study were obtained from MEASURE DHS [18] and from FNRI [19]. Full review of this study from an institutional review board was not sought as the datasets were anonymous and they are available for public use with no identifiable information on the survey participants.

### Aim and study concept

This study aims to quantify the cost of MNDs in the Philippines by comparing the current nutritional situation with a hypothetical scenario in which all children have a sufficient MN intake, either through their diet or through MN supplementation. We build a health economic model simulating the consequences of MNDs in childhood over the entire lifetime. Costs of MNDs in adulthood are only considered if they are the consequences of childhood micronutrient malnutrition. The model is referenced to the year 2008 for which we have the results of a major health survey, the 2008 National Demographic and Health Survey (DHS) [18], and of a major nutrition survey, the 2008 National Nutrition Survey (NNS) [19]. The model was developed both in Microsoft Excel and R and its technical details are described in Additional file 1.

### Study design

**Figure 1:**
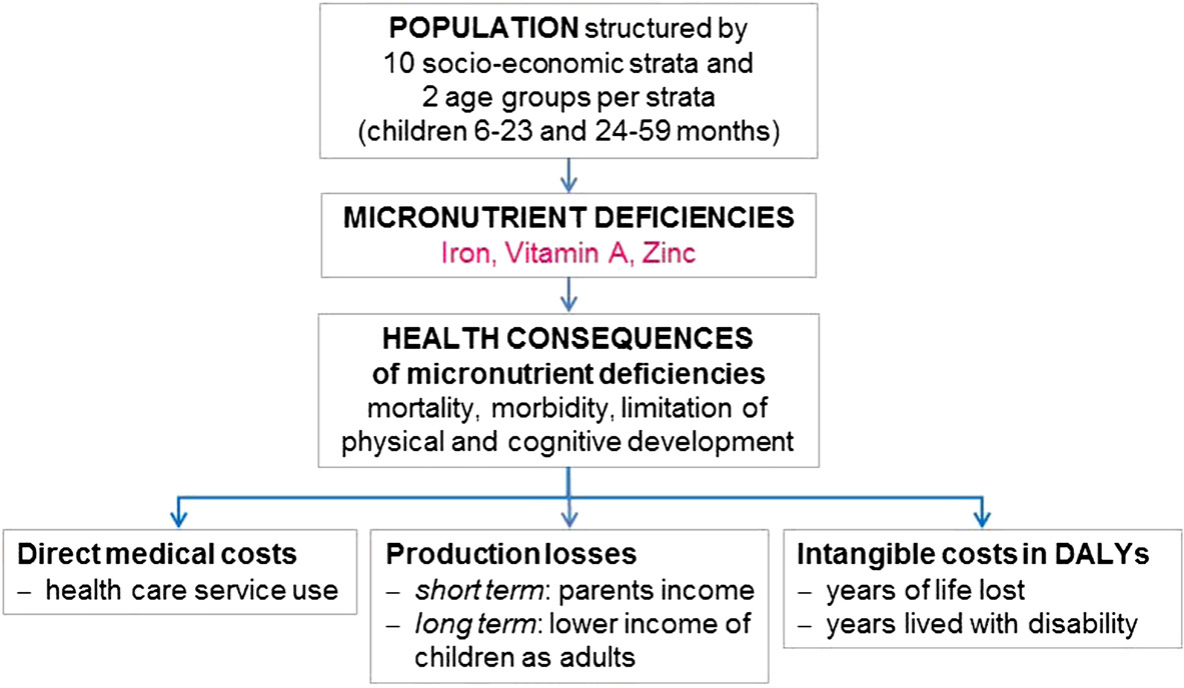
**General structure of study.** The DHS and NNS sample populations are first structured into 10 socio-economic strata (SES) and 2 age groups of children per SES (6–23 months and 24-59 months). In a second step the MNDs of children are assessed in each of these groups. In a third step the lifetime health consequences of these MNDs are modelled based on information extracted from literature. In a final step the direct medical costs, production losses and intangible costs caused by these health consequences are computed and long-term costs are discounted to present value.

The study follows a bottom-up, incidence-based design with a societal perspective and a lifetime horizon. *Bottom-up* means that costs are first calculated on the data collected from single households contacted in the 2008 DHS and NNS surveys and then extrapolated to the total population. *Incidence-based* means that the model computes the lifetime costs for a one-year cohort of children 6–17 months old in 2008. *Societal perspective* means that all the costs occurring in the society are considered. These costs include disease related health care expenditures (direct medical costs), current and future income losses of individuals of working age due to absence from work and reduced productivity (production losses) and quality and quantity of life years lost (intangible costs) (Figure 1).

### Study population

Our model differentiates 6-59-month-old children by 10 socio-economic strata (SES) (1 = lowest decile of households, 10 = highest decile) and two age groups (6–23 months and 24–59 months). Levels of MNDs as well as their consequences are particularly severe in these age groups as the body stores of micronutrients tend to be progressively exhausted at the age of 6 months and the child’s diet is often not adequate to meet the great need for micronutrient intake in periods of rapid growth. Although children below this age range may also suffer from MNDs [20] they are excluded from the analysis as our study is part of a more extensive project focussing on nutritional interventions for 6-59-month-old children. Furthermore, exclusive breast feeding is recommended up to 6 months of age [21]. Meanwhile, children older than 59 months are excluded from the analysis as they have a reduced risk of MNDs [22].

Both the DHS [18], carried out by the Philippines National Statistics Office, and the NNS [19], carried out by the Philippines Food and Nutrition Research Institute (FNRI), are large and representative population surveys with 12,469 respectively 36,634 households successfully contacted in 2008. We had access to the complete NNS and DHS [23] data. Households from the two surveys were stratified by deciles into 10 SES. SES were defined by a wealth index similar to the conventional DHS wealth index [24] but based only on household characteristics contained in the NNS as well as in the DHS.

We use the following terminology for the populations considered: The *sample population* consists of the 6–59 months old children from the DHS and NNS. The *model population* is the sample population extrapolated to the total population representing all 6-59-month-old Filipino children in 2008. The *model cohort* is the 1-year-cohort of 6-17-month-old children from the model population for which lifetime cost consequences of MNDs are calculated. Costs are calculated separately for MNDs at the age of 6–23 months and 24–59 months.

### Prevalence of MNDs

The MNDs considered in this study are those of iron, vitamin A and zinc, as the health consequences of these deficiencies are well documented. We exclude iodine deficiency, although its health consequences are also well documented, because iodine fortification is focused on salt as a carrier [25] and not on milk and cereals, which will be the fortified foods considered in the third part of our study. The study ignores the health consequences of iron-deficiency in non-anemic children.

Information on the prevalence of MNDs was extracted from the 2008 NNS for the 2 age groups over the 10 SES. Prevalence rates are calculated by assuming a normal distribution defined by the mean and the standard deviations (SD) of the blood values provided by FNRI and applying the cut-off levels reported in Table 1. The probability of being below the cut-off level is equal to the prevalence rate of MNDs in the population. Prevalence of anemia was calculated as the proportion of children with hemoglobin level below WHO cut-off levels [26] for moderate and severe anemia (Table 1). 60% of cases of anemia were attributed to iron deficiency anemia (IDA) [27]. Vitamin A deficiency (VAD) was measured with serum retinol level and zinc deficiency (ZnD) with serum zinc level [19]. Serum zinc has been criticized as a measure of ZnD, as ZnD may be present even if the serum level is in the normal range. Nonetheless serum zinc is the best known measure of deficiency and has been shown to be a reasonable indicator of deficiency at a population level [28].

**Table 1:** Cut-off levels for MNDs

**Micronutrient deficiency**	**Blood value**	**Cut-off level**	**Source**
Iron deficiency anemia	Hemoglobin	100 [g/L] for moderate and 70 [g/L] for severe anemia	[26]
Vitamin A	Serum retinol	20 [μg/dL]	[3]
Zinc	Serum zinc	65 [μg/dL]	[19]

Information on blood values were obtained from FNRI as mean and SD for each of the 20 groups of children. Although this information is mostly based on reasonable sample sizes of up to 250 children per group, some groups have a small sample size with only 15 observations in the smallest group. In order to be able to extrapolate the sample data to the whole population, without magnifying uncertainty due to limited sample size, we smoothed the distribution of these group means. The levels of MNDs in the 20 groups correspond to the average group specific deficiency levels measured in the NNS.

### Attribution of health consequences

Whenever possible, the attribution of health consequences to MNDs is based on systematic reviews of the effects of MN supplementation trials for children aged 6–59 months. We assume that MN supplementation trials were designed to fill the gap of MN intake (e.g. in terms of adequate intake) due to the insufficient diet of the children and thus eliminate all the adverse health consequences of MNDs, an approach also applied in previous research [29]. The health consequences of MNDs thus correspond to the difference in health status of the intervention and the control group in the supplementation trials.

Some of the health effects of MNDs are calculated by attributing a fraction of the current prevalence of diarrhea and lower respiratory diseases to a specific micronutrient. We calculate the population attributable fractions (PAF) based on relative risks (RR) extracted from the literature by applying Levin’s classical formula [30]. These PAF are then applied to the current mortality and prevalence of relevant diseases in order to calculate the number of episodes attributable to MND. Prevalence of measles is not covered by DHS and therefore estimated using the nationwide prevalence rates and the immunization rates by group according to DHS data. Child mortality rates were calculated according to the procedure used in the DHS report [18]. The influence of MNDs on mortality is modeled as influence on all-cause mortality. The effect may be direct via diseases associated with MNDs (diarrhea, lower respiratory disease, and measles) or indirect via increased incidence of other diseases.

### Attribution of costs to health consequences

Attribution of costs to the health consequences of MNDs is based on information extracted from the DHS, literature and other sources. These costs include costs in the immediate occurrence of the disease as well as long-term costs due to the lifetime consequences of impaired physical and mental development. Intangible costs are measured in DALYs, while monetary costs are measured in US Dollars (USD). Quantities originally measured in Philippine Pesos (PHP) were converted into USD using an average 2008 exchange rate.

Calculation of *direct medical costs* is mainly based on the DHS [18]. The households interviewed in the survey were asked for the costs borne in the treatment of diarrhea and lower respiratory infections of their children, which are both frequent adverse health consequences of MNDs. We assume that the specified costs in the DHS represent out-of-pocket expenditure of the households. As health service providers in the Philippines are in part financed by government agencies, we increase these out-of-pocket expenditures with a factor corresponding to the public share in overall health expenditures [31,32].

*Production losses* are calculated according to the human capital approach and thus correspond to the current and future gross monetary income losses. They include income losses of parents who cannot work because they have to take care of their sick children and the lifetime income losses due to impaired physical and cognitive development and premature death of children. We assume that income losses of parents only occur in households in which both parents work. Whereas current production losses are valued with SES specific incomes, future losses are all valued with an average income. In order to account for future economic growth, we assume a yearly increase in wages of 1.66%, corresponding to the average growth rate of real GDP per capita in the years 1990–2011 [33].

*Intangible costs* are calculated as Disability Adjusted Life Years (DALYs) lost without applying age weights [34]. DALYs are lost due to current illness, premature death and future permanent disabilities. We do not quantify DALY losses in monetary terms, as this is often criticised as ethically and methodologically questionable [35].

Production losses and intangible costs can be considered as two separate and non-overlapping dimensions of the costs of MNDs as recent research has shown that double counting of losses considered in both cost dimensions is negligible [36,37].

### Discounting future losses to present value

**Figure 2:**
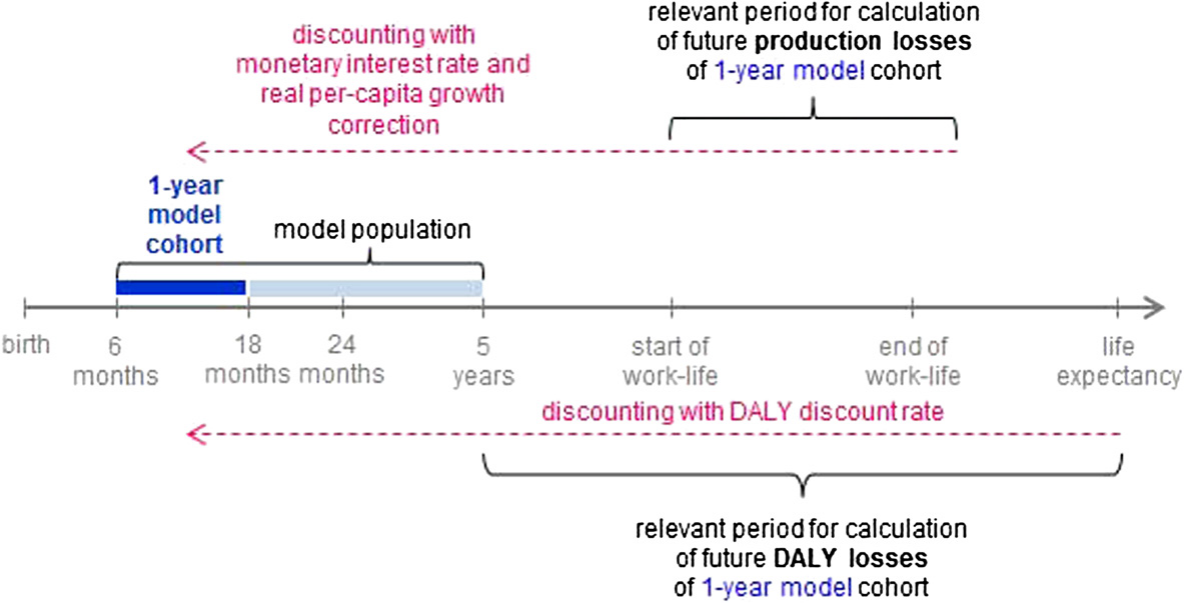
**Simplified representation of relevant time periods for future costs.** Future losses occurring in the 1-year model cohort are discounted to present value taking account of the time frames in which the losses occur.

Future production losses have to be discounted to their present value because a dollar today is worth more than a dollar in 20 or 30 years (Figure 2). We apply a discount rate of 3% which is a widely accepted value in cost-of-illness studies [38].

For discounting of future life years we follow the Global Burden of Disease Report [39], which discounts future DALY losses with a 3% rate in the baseline analysis but reports undiscounted DALYs as well (as in the recently published 2010 GBD [40]).

Discounting takes account of the different time periods in which losses accrue. While future DALYs lost are calculated from the time of event to the end of expected lifetime, future production losses occur only during work-life, starting at the age of labor force participation and ending with the end of work-life. Total costs of the model can thus be interpreted as the present value of all lifetime losses of the 1-year model cohort.

### Sensitivity analysis

In order to test the robustness of our results and to understand the influence of single model parameters we run three types of sensitivity analysis (SA). *First*, we carry out a *deterministic SA* by observing how results change when each of the single model parameters is modified by minus and plus 20% compared to the base values. This type of analysis allows us to estimate the importance of single parameters for model results. *Second*, we explore consequences of modifications in two key assumptions, namely the future wages, and the effect of ZnD on mortality. *Third*, we carry out a *probabilistic SA* by randomly varying all model parameters within predefined distributions and then running the model 10,000 times. The probabilistic SA allows us to explore the interactions between the model parameters and to evaluate a much larger range of parameter variations than the deterministic SA. Table 2 reports the types of distribution assumed for the single categories of model parameters. Where information on the variability of the parameters is not available from the literature, we choose a SD equaling 20% of the mean.

**Table 2:** Assumptions on distributions of model parameters in probabilistic SA

**Parameter**	**Distribution**
Unrestricted parameters, such as cognitive score difference due to IDA	Normal
Non-negative parameters such as duration of illnesses and risk estimates	Lognormal
Parameters with 0,1 range, such as disability weights and share of IDA	Beta
Cost parameters	Gamma

## Results

### Model Population

The model population consists of all Filipino children aged 6–59 months stratified by 10 SES and 2 age groups. This population is modeled on the basis of the DHS 2008 population (60,901 individuals in 12,469 households) and extrapolated to the total population of 90,354,753 individuals [41]. Table 3 reports the estimated number of children per SES and age group. The 9 million children are unevenly distributed among the SES as poor households have a substantially higher number of children than the wealthier households.

**Table 3:** Model population (in 1,000 children) by SES and age group

**Socio-economic strata**	**1 low**	**2**	**3**	**4**	**5**	**6**	**7**	**8**	**9**	**10 high**	**Total**
Children aged 6–23 months	403	348	344	354	310	271	299	265	233	234	3,061
Children aged 24–59 months	832	790	737	642	589	573	561	493	475	406	6,098
Percentage share of SES on total of children 6–59 months	13.5	12.4	11.8	10.9	9.8	9.2	9.4	8.3	7.7	7.0	100.0

Source: [18,41], own calculation.

Table 4 shows child mortality and income by SES. Child mortality in the lowest and the highest SES differ by a factor of 3 for the children aged 6–23 months and by a factor of 17 for the children aged 24–59 months. Income distribution is strongly skewed towards the higher SES with over half of income earned by the top 2 SES [42].

**Table 4:** Mortality and income distribution by SES

**Socio-economic strata**	**1 low**	**2**	**3**	**4**	**5**	**6**	**7**	**8**	**9**	**10 high**	**Total**
Death per year per 1,000 children aged 6–23 months	10.2	8.2	7.0	6.1	5.5	4.9	4.5	4.1	3.8	3.5	57.7
Death per year per 1,000 children aged 24–59 months	3.5	2.8	2.2	1.7	1.2	0.9	0.6	0.4	0.2	0.2	13.6
Share of total income of SES on total income	1.8	2.9	3.7	4.6	5.7	7.1	9.0	11.8	16.6	36.8	100

Source: [18,42], own calculation.

### Prevalence of MNDs

**Figure 3:**
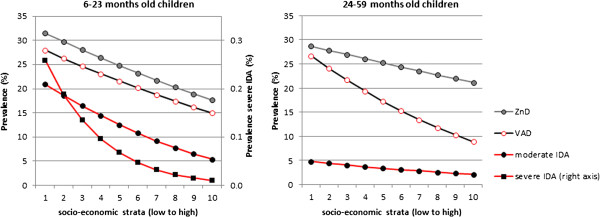
**Prevalence of MNDs by SES and age group.** Source: [36], own calculation. No severe IDA in the 24–59 months age group.

The prevalence of IDA, VAD and ZnD in the model population is calculated on the basis of hemoglobin, serum retinol and serum zinc values measured in the NNS [19] (Table 5 and Figure 3). Overall prevalence of MNDs in the age group of 6-23-month-old children is 13% for IDA, 22% for VAD and 25% for ZnD. All MNDs are markedly more prevalent in the lower SES. With a 4 times higher level of IDA in the poorest than the wealthiest SES, this difference is particularly evident for moderate IDA among 6-23-month-old children. The contrast between SES is even stronger for severe IDA, which is, however, relatively rare even in the children living in the poorest households. Moderate IDA is substantially lower in the group of 24-59-month-old children and there is virtually no severe IDA in this age group. For VAD there is a twofold difference in prevalence between the lowest and the highest SES. Prevalence decreases only slightly as children grow older, and this decrease is more marked among the children living in wealthier households. ZnD has the highest overall prevalence but differs less between high and low SES and age groups.

**Table 5:** Prevalence of MNDs by SES (%)

		**Socio-economic strata (SES)**										
	**Age group (months)**	**1 low**	**2**	**3**	**4**	**5**	**6**	**7**	**8**	**9**	**10 high**	**Overall mean**
**Moderate IDA**	6-23	20.92	18.67	16.51	14.47	12.57	10.82	9.23	7.80	6.54	5.42	13.13
	24-59	4.81	4.43	4.07	3.73	3.42	3.12	2.85	2.60	2.35	2.14	3.54
**Severe IDA**	6-23	0.26	0.19	0.14	0.10	0.07	0.05	0.03	0.02	0.02	0.01	0.10
**VAD**	6-23	27.99	26.31	24.68	23.11	21.62	20.17	18.80	17.46	16.19	15.00	21.82
	24-59	26.68	24.10	21.66	19.38	17.25	15.28	13.46	11.80	10.28	8.91	18.11
**ZnD**	6-23	31.51	29.76	28.06	26.42	24.82	23.27	21.78	20.35	18.97	17.65	25.00
	24-59	28.72	27.82	26.94	26.08	25.25	24.39	23.57	22.76	21.97	21.20	25.39

Source: Own calculation, based on [19].

Overall mean is higher than mean of SES prevalence due to higher number of children in poorer households. No severe IDA in the 24–59 months age group.

### Health and cost consequences of MNDs

**Figure 4:**
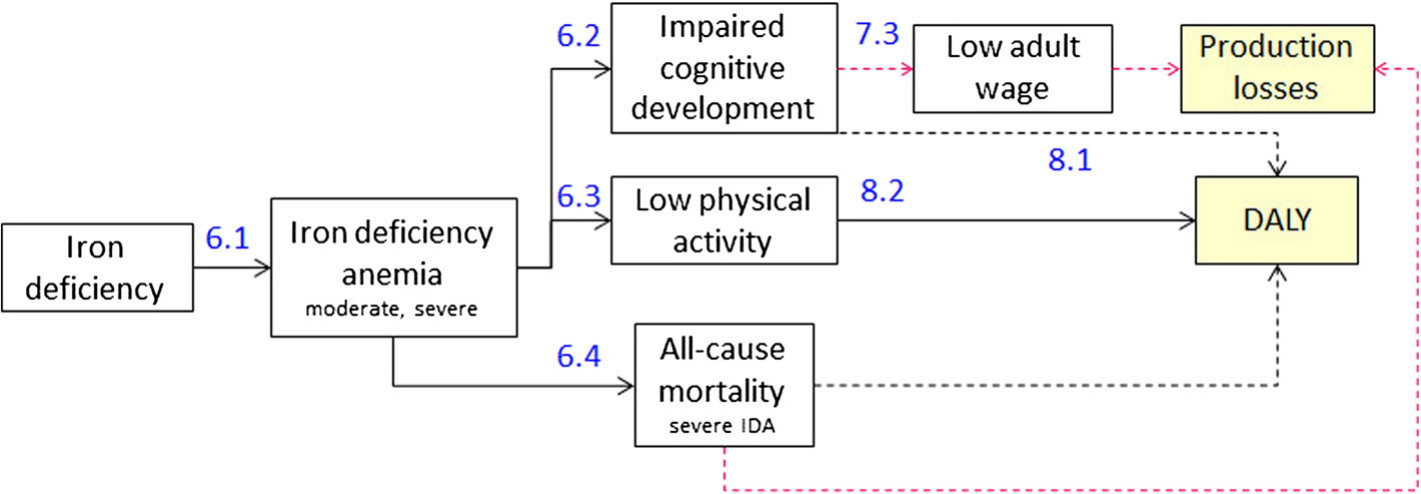
**Health and cost consequences of IDA.** Numbers refer to source of information of effect size or DALY weight in Tables 6, 7 and 8 (first number identifies table). Dotted lines represent long term consequences.

**Figure 5:**
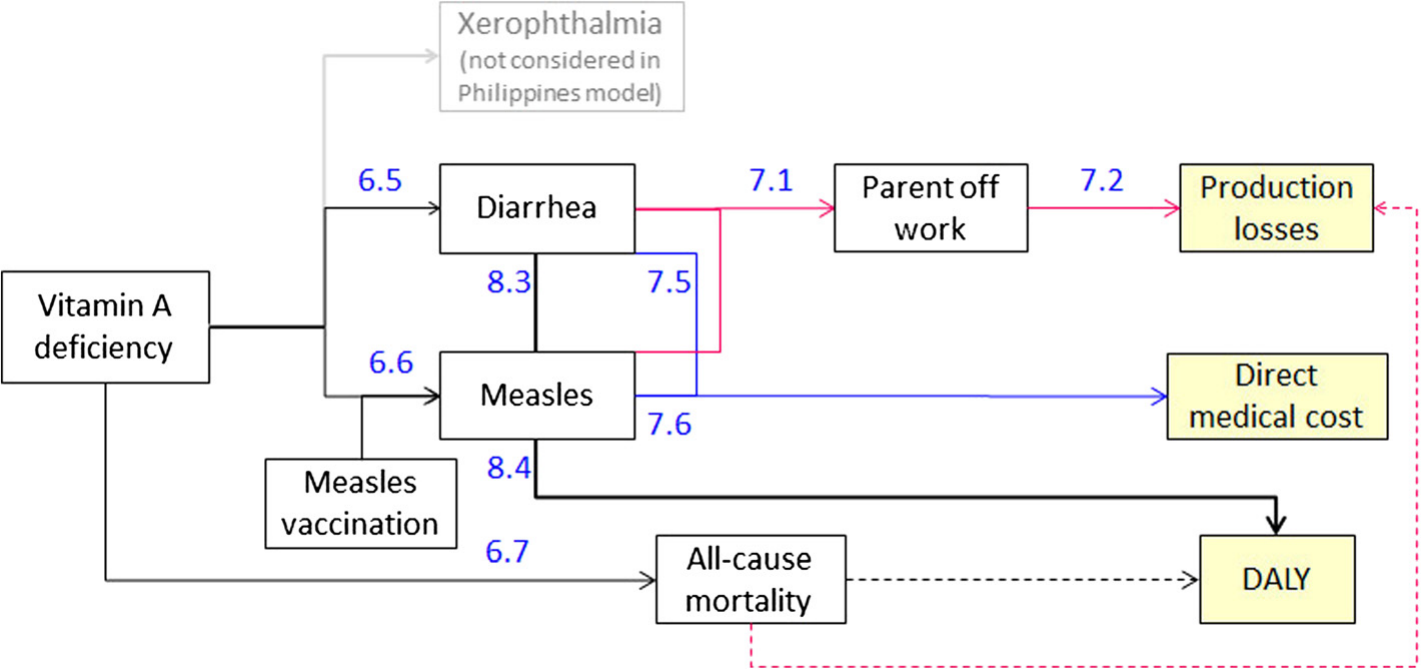
**Health and cost consequences of VAD.** Numbers refer to source of information of effect size or DALY weight in Tables 6, 7 and 8 (first number identifies table). Dotted lines represent long term consequences.

**Figure 6:**
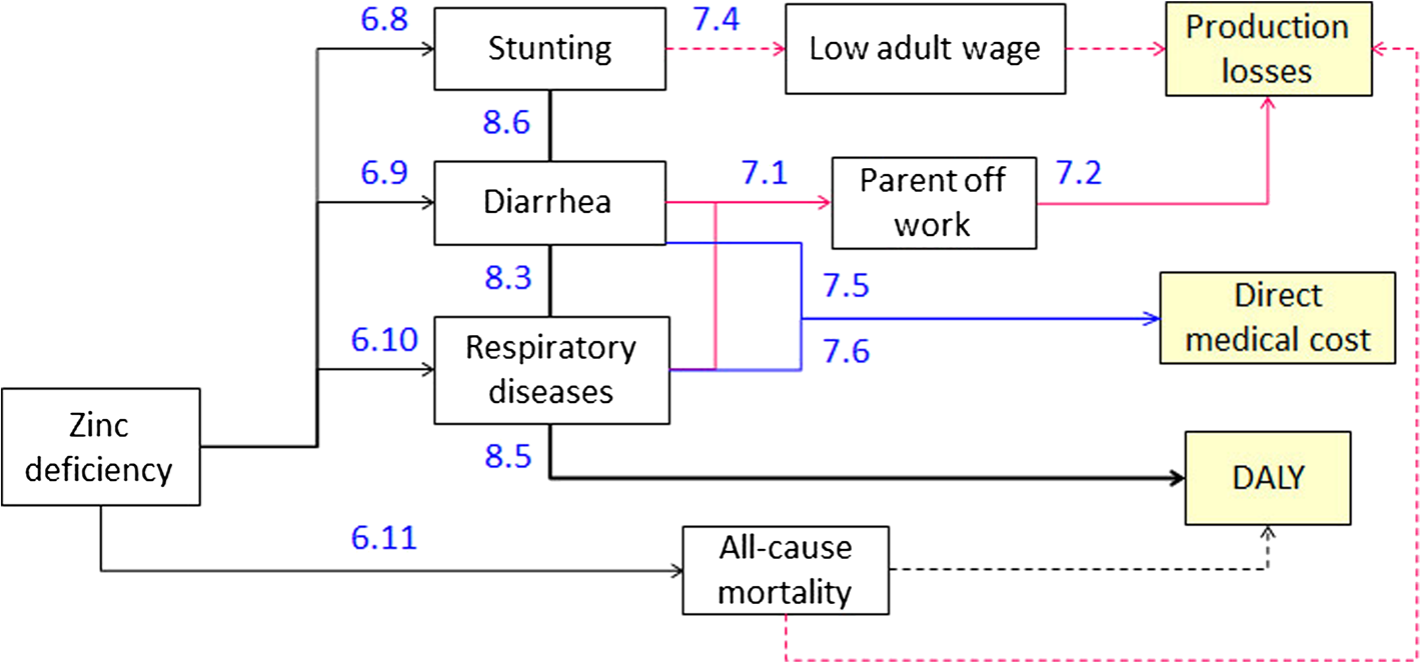
**Health and cost consequences of ZnD.** Numbers refer to source of information of effect size or DALY weight in Tables 6, 7 and 8 (first number identifies table). Dotted lines represent long term consequences.

The epidemiological frameworks of adverse health consequences due to IDA, VAD and ZnD (Figures 4, 5, and 6) are based on an extensive literature review completed by repeated consultations with the experts of our advisory board. Wherever possible we drew information from published systematic reviews of MN supplementation trials.

Tables 6, 7, and 8 show the effect sizes of the single adverse health consequences and the dimension of related costs and disability weights. Connecting arrows in Figures 4, 5, and 6 are labeled with numbers referring to information contained in the Tables 6, 7, and 8 (table and row number).

**Table 6:** Health consequences of MNDs

**MND**	**Health consequence**	**Effect size (SD where available)**	**Persistent effect**	**Numbering in Figures 4, 5 and 6**	**Source**	**Source systematic review**
IDA	Share of anemia caused by iron deficiency	60%	Not applicable	6.1	[27,51]	No
	Cognitive impairment and motor development	Moderate or severe IDA in 6-23-month-old children: Cognitive score in adults reduced by 9 points (on a scale with mean 100 and SD 15)	Yes	6.2	[43]	No
	Low physical activity	Moderate or severe IDA: All children	No	6.3	[39,46]	No
	All-cause mortality	Moderate IDA: No effects	Yes	6.4	[52]	Yes
		Severe IDA: RR of 2.21				
VAD	Diarrhea	RR of 1.18 (1.15, 1.22)	No	6.5	[47]	Yes
	Measles	RR of 2.00 (1.49, 2.70)	No	6.6	[47]	Yes
	All-cause mortality	RR of 1.32 (1.20, 1.45)	Yes	6.7	[47]	Yes
ZnD	Stunting	Children 24–59 months: Height for age z-score is reduced by 0.17	Yes	6.8	[15]	Yes
	Diarrhea	RR of 1.25 (1.11, 1.41)	No	6.9	[15]	Yes
	Respiratory diseases	RR of 1.18 (1.03, 1.33)	No	6.10	[15]	Yes
	All-cause mortality	RR: 0.95 (0.81, 1.11). The model thus assumes no effect of ZnD on mortality.	Yes	6.11	[15]	Yes

Health consequences are assumed to be valid and equal for both age groups, with the exception of health impairments due to ZND and cognitive impairment due to IDA.

**Table 7:** Cost consequences adverse health effects

		**Effect size**	**Numbering in Figures **4**,**5** and **6	**Source**
**Productivity losses**				
Current	Diarrhea, measles and respiratory disease	2 days off work for 1 parent per episode of illness (only if both work)	7. 1	Assumption
	Parent off work	Loss of daily gross wage of USD 1.1 -23.2 depending on SES	7.2	[53]
Future	Impairment cognitive development	1 SD reduction of the cognitive score leads to a reduction of adult wage by 8%	7.3	[8,44]
	Stunting	1% decrease in height leads to a reduction of adult wage by 9.23% (average height in the Philippines is 158 cm)	7.4	[19,48,49,54]
Treatment costs per episode*				
Current	Diarrhea	9.6 USD	7.5	Calculation on DHS [18] and PNHS [32]
		Only complicated cases are treated (41% of cases in DHS)		
	Measles	92.7 USD	7.6	Calculation on DHS [18], WHO [55] and PNHS [32]
		By assumption only complicated cases are treated and treatment costs are equal to those of respiratory diseases (75% of cases) [56].		
	Respiratory disease	92.7 USD	7.7	Calculation on DHS [18] and PNHS [32]
		Only complicated cases are treated (60% of cases in DHS)		

* Costs covered by health insurance companies and government agencies amount to 49% in addition to out-of-pocket expenditures by households registered in DHS.

**Table 8:** Disability weights of adverse health effects

**Health consequence**	**Disability weight**	**Effect duration**	**Numbering in Figures **4**, **5** and **6	**Source**
Cognitive impairment	Moderate IDA: 0.006	Permanent	8.1	[47]
	Severe IDA: 0.024			
Low physical activity	Moderate IDA: 0.011	Temporary	8.2	[47]
	Severe IDA: 0.087			
Diarrhea	0.119	Temporary	8.3	[39]
Measles	0.152	Temporary	8.4	[39]
Respiratory disease	0.279	Temporary	8.5	[39]
Stunting	0.002	Permanent	8.6	[39]

For ZnD and VAD they are obtained from the global burden of disease study by the WHO [39], while weights for IDA are provided by Stein, who distinguishes weights by type of disability [46].

IDA leads to impaired cognitive development, low physical activity and, in case of severe IDA, to increased all-cause mortality (Figure 4). 60% of anemia measured in the population is defined as IDA [27]. The impact on cognitive development of 6-23-month-old children leads to a substantial reduction of cognitive score in adult age and a consequent loss in wages earned [8,43-45]. DALY losses are calculated with the disability weights proposed by Stein [46] as they distinguish between short term effects on low physical activity and the long term effects of impaired cognitive development. Increased all-cause mortality due to severe IDA leads to production losses and DALY losses as the whole lifetime and income of the children is lost.

VAD leads to a rise in the number of cases of diarrhea and measles and increased all-cause mortality [47]. We exclude xerophthalmia and blindness, because these consequences of severe VAD are no longer observed in the Philippines (expert knowledge of authors affiliated to Philippines Food and Nutrition Research Institute).The attribution of the number of cases of measles due to VAD takes account of the SES specific rates of measles vaccination. We assume that, in households where both parents are working, diarrhea and measles lead to an absence from work of one of the parents for 2 days and the consequent loss of income. Diarrhea and measles also lead to direct medical costs and temporary DALY losses. Increased all-cause mortality leads to long term production and DALY losses.

ZnD leads to a rise in the number of cases of diarrhea and respiratory disease, stunting and increased all-cause mortality. Cost consequences of diarrhea and respiratory disease are calculated as for VAD. The effect of ZnD on mortality is unclear. An extensive review finds a significant reduction in mortality risk when zinc is given alone but no significant reduction when given in combination with other nutrients [15]. We thus exclude an effect of ZnD on mortality in our analysis. Stunting leads to lower adult wages and DALY losses. The estimated effect of stunting on adult wage varies widely with an additional centimeter of adult height leading to an increase in wages between 0.86% and 15.8% [48]. We base our estimate on Gao and Smyth [49] which use more recent data than other studies [48] and implement a validated estimation strategy [50].

### Costs of MNDs

Total lifetime costs of iron, vitamin A and zinc deficiencies in 6-59-month-old Filipino children amounted to direct medical costs of 30 million dollars, production losses of 618 million dollars and intangible costs of 122,138 DALYs (Table 9). These costs can be interpreted as the lifetime costs of a 1-year cohort affected by MNDs between the age of 6–59 months. The table also reports the costs if the cohort is affected by MNDs only between the age of 6–23 months *or* between 24–59 months.

**Table 9:** Total costs by cost category, MND, reference period (current, future) and age group

** MND**	**Medical cost (million USD)**	**Production losses (million USD)**				**Intangible costs (DALYs)**				**Number of premature deaths**
		**Within age group**	**Age > 5 years**	**Death**	**Total**	**Within age group**	**Age > 5 years**	**Death**	**Total**	
**Costs due to MNDs in 6–23 months old children**										
Iron			384.3	0.9	**385.1**	5′918	47′277	841	**54′036**	28
Vitamin A	1.8	0.5		38.7	**39.2**	394		37′976	**38′370**	1′284
Zinc	8.5	0.7		0.0	**0.7**	982		0	**982**	0
**Total**	**10.3**	**1.2**	**384.3**	**39.6**	**425.1**	**7′294**	**47′277**	**38′817**	**93′389**	**1′313**
**Costs due to MNDs in children 24–59 months**										
Iron						2′373		6.8	**2′380**	0
Vitamin A	1.5	0.3		19.8	**20.2**	327		18′745	**19′073**	632
Zinc	18.5	1.1	171.8	0.0	**173.0**	2′032	5′264	0	**7′297**	0
**Total**	**20.0**	**1.5**	**171.8**	**19.8**	**193.1**	**4′733**	**5′264**	**18′752**	**28′749**	**632**
**Costs due to MNDs in children 6–59 months**										
Iron			384.3	0.9	**385.1**	8′291	47′277	848	**56′416**	29
Vitamin A	3.3	0.8		58.6	**59.4**	722		56′721	**57′443**	1′923
Zinc	27.0	1.9	171.8	0.0	**173.7**	3′014	5′264	0	**8′279**	0
**Total**	**30.3**	**2.7**	**556.1**	**59.4**	**618.2**	**12′027**	**52′542**	**57′569**	**122′138**	**1′951**

Costs age > 5 years and death at present value (3% discount rate).

Direct medical costs for treatment of diarrhea, respiratory disease and measles are dominated by costs due to ZnD (89% of total). Production losses are dominated by losses in future lifetime (90% of total), due to impaired mental and physical development, and premature death (10%) while current losses due to the absence of parents from work when caring for their sick child appear to be of minor importance (<1%). Intangible costs are mainly due to premature death (47%) followed by future lifetime losses (43% of total DALY losses) and losses in the current year (10%).

Costs of IDA are mainly due to its impact on mental development in 6-23-month-old children (100% of production and 84% of DALY losses). The additional effect of IDA in 24-59-month-old children is limited to DALY losses due to reduced physical activity. As IDA has no relevant direct effect on other diseases, it does not lead to medical costs (medical costs of all-cause mortality are not considered in the model). Costs of VAD are predominately caused by increased mortality of children in both age groups (99% of production and 99% of DALY losses). The main cost-effect of ZnD is on future production losses due to stunting which amounts to 28% of total production losses.

Costs of MNDs differ considerably between SES (Table 10) as costs in the poorest third of the households are 5 times higher than in the wealthiest third. These differences are a result of both higher prevalence of MNDs in lower SES as well as a higher number of young children in poor households.

**Table 10:** Distribution of costs over SES by MND and cost category

		**Socio-economic strata**										
**MND**	**Cost category**	**1 low**	** 2**	** 3**	** 4**	** 5**	** 6**	** 7**	** 8**	** 9**	** 10 high**	** Total**
**IDA**	Production losses	82.44	62.81	51.93	54.34	37.13	28.26	23.38	19.62	13.45	11.76	**385.12**
	DALYs lost	12,334	9,306	7,653	7,777	5,361	4,086	3,410	2,823	1,967	1,699	**56,416**
**VAD**	Medical costs	0.72	0.57	0.47	0.41	0.31	0.24	0.21	0.16	0.12	0.10	**3.30**
	Production losses	17.28	11.62	8.59	6.51	4.55	3.22	2.77	2.00	1.49	1.32	**59.4**
	DALYs lost	16,802	11,277	8,349	6,327	4,412	3,119	2,675	1,914	1,400	1,169	**57,443**
**ZnD**	Medical costs	5.66	4.66	3.88	3.32	2.53	2.08	1.74	1.34	1.02	0.75	**26.98**
	Production losses	20.39	26.05	22.06	21.31	18.92	18.20	13.76	12.34	11.26	9.40	**173.70**
	DALY lost	1,185	1,281	1,093	1,017	868	801	631	544	475	382	**8,279**
**All**	Medical costs	6.37	5.23	4.35	3.73	2.84	2.32	1.95	1.50	1.14	0.85	**30.28**
	Production losses	120.12	100.48	82.58	82.16	60.60	49.68	39.92	33.96	26.20	22.48	**618.18**
	DALY lost	30,321	21,864	17,095	15,120	10,642	8,006	6,717	5,282	3,842	3,249	**122,138**

Medical cost and production losses in million USD, future costs and DALY losses at present value (3% discount rate).

### Results of sensitivity analysis

**Figure 7:**
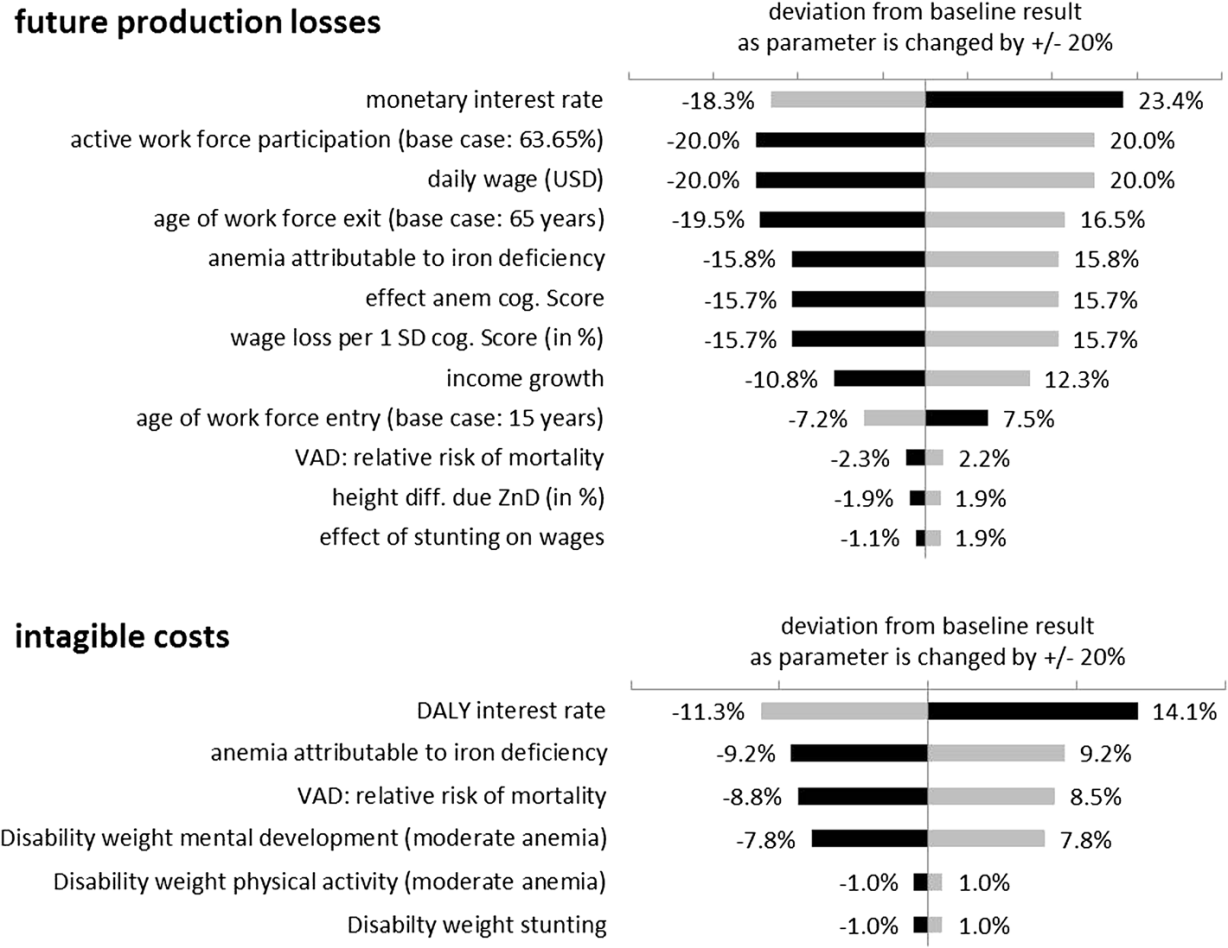
**Factors with strongest influence on intangible costs and future production losses.** Grey bars indicate the consequence of an increase of the parameter by 20%; black bars the effect of a decrease of 20%.

In the deterministic sensitivity analysis (SA) we first record how results change as single model parameters are increased or diminished by 20% and then summarize these results in tornado diagrams, by ordering them from the largest to the smallest effect. Figure 7 displays the tornado diagrams for future production losses and intangible costs for all the parameters that change the outcome by at least 1%.

Future production losses are most affected by the discount rate applied to future losses. Changes in the factors influencing labor market participation and wage level have an almost one-to-one effect on production losses. Further important factors are the parameters with an impact on impaired cognitive development induced by IDA.

Intangible costs are most affected by a change of the discount rate applied to future DALY losses, as the present value of DALYs far away in time is strongly reduced by discounting. For example 70 life years in full health lost, equal to 70 DALYs lost, correspond to 29 DALYs at present value when discounted with a 3% rate. Without discounting DALYs lost in future lifetime due to impaired development and premature death would increase by 138% and total DALYs by 125%. Intangible costs are also strongly affected by changes in the parameters influencing the lifetime costs of IDA and the relative risk of mortality due to VAD.

Due to the high uncertainty regarding future wages, we chose to evaluate two alternative scenarios: 1) Future earnings corresponding to current SES specific earnings. 2) Future earning corresponding to median income (median income is lower than mean income as distribution of earnings is highly skewed with 53% of income in the upper 20% of households). In both scenarios productivity losses are considerably reduced. Overall productivity losses decrease by 37% in scenario 1 and by 36% in scenario 2.

The systematic review that is the source of the effects of ZnD on health provides two estimates of relative risks for mortality. In the base case we use the estimate from supplementation trials providing zinc in conjunction with other micronutrients. For the sensitivity analysis we also run our model using information on the RR derived from supplementation trials where only zinc is tested against a placebo. Based on these studies, the authors find a RR for mortality of 1.22 [15]. When applying this RR of mortality in our model, DALYs lost would increase by 30% and production losses by 6%.

**Figure 8:**
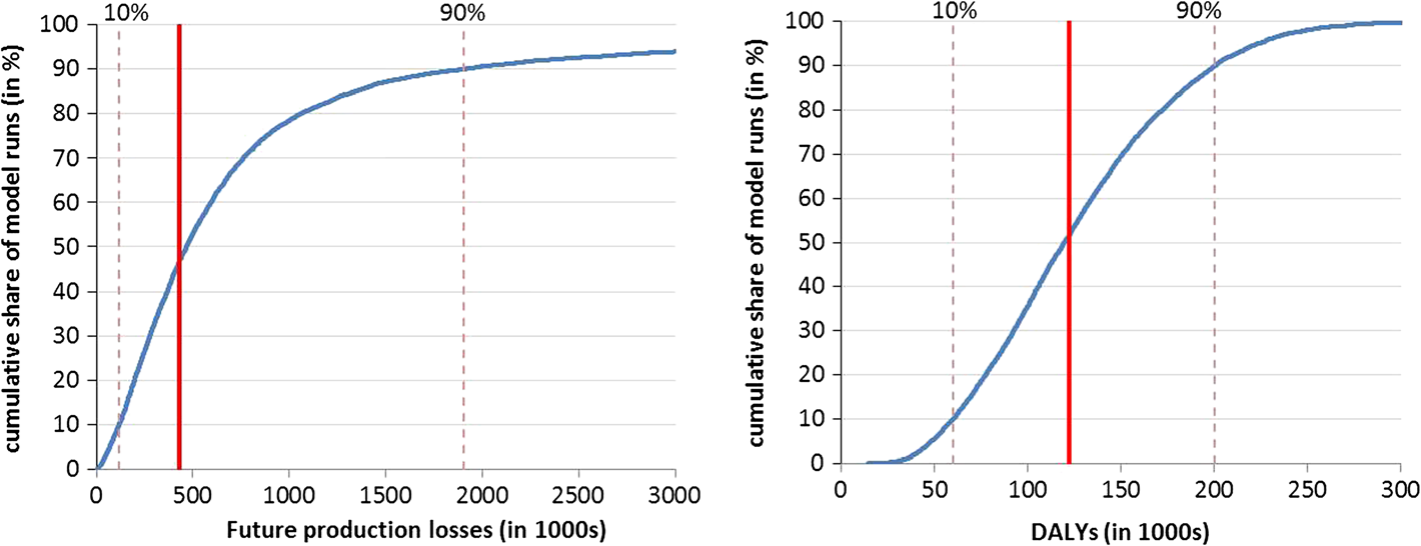
**Results of probabilistic SA for future production losses and intangible costs.** The curves indicate the cumulative distribution of future production losses and intangible costs. The solid vertical line represents the main results of the model while the dotted vertical lines delimit the 80% confidence interval of the results of the probabilistic SA.

Probabilistic SA evaluates the overall variability of the results by randomly varying all model parameters in 10,000 model runs. Figure 8 shows that intangible costs were between 62,000 and 194,000 DALYs in 80% of the model runs. Future production losses lay between 161 and 2,531 million dollars in 80% of the model runs, with a distribution skewed to the right due to the shape of underlying parameter distributions.

## Discussion

We present a health economic model simulating the lifetime cost consequences of iron, vitamin A and zinc deficiencies in 6-59-month-old children in the Philippines stratified in 10 SES and 2 age groups. The model includes a detailed representation of the consequences of MNDs on health and physical and mental development. Total lifetime costs amount to medical costs of 30 million dollars, production losses of 618 million dollars and intangible costs of 122,138 DALYs. Costs are dominated by future lifetime costs due to impaired mental and physical development (90% of production and 43% of DALY losses) and costs of premature deaths (10% of production and 47% of DALY losses) over current losses. Burdens of MNDs differ substantially between SES as the costs in the poorest third of the households are 5 times higher than in the wealthiest third. Intangible costs are considerably higher for VAD and IDA than for ZnD while direct medical costs are dominated by ZnD. Production losses on the other hand are mainly determined by IDA, followed by ZnD. Nearly all the costs of MNDs in the Philippines are incurred by households, as they sustain a large share of medical costs and of the income lost by parents looking after their sick children and, most importantly, the huge income and DALY losses borne by children in their future lifetime.

### Magnitude of costs

It may be helpful to examine the magnitude of these results. The 122,138 DALYs lost are driven by the premature death of 1,951 child deaths corresponding to 57,569 DALYs lost. If we compare total DALYs lost with DALYs lost due to premature death, we may conclude that the equivalent of 4,000 complete lifespans are lost each year because of MNDs. Comparing our undiscounted results with the total undiscounted 22.2 million DALYs lost in the Philippines in 2010 according to the GBD [57] the share of DALYs lost due to MNDs amount to 1.24% of total DALYs lost. Health consequences include 5.6 million days of diarrhea, 1.5 million days of respiratory disease and 346,331 stunted children. Total monetary costs (direct medical costs and production losses) amount to 648 million dollars corresponding to 0.37% of GDP or 9.5% of total private and public health care expenditures in the Philippines in 2008 [58]. Although direct medical costs appear low in comparison to the other cost categories, they can be substantial for poor households. The out-of-pocket expenditure for the treatment of an episode of diarrhea and lower respiratory disease, for example, amount to 6 and 62 dollars respectively while average weekly income of the poorer half of households is at 11 dollars.

### Strengths and limitations

To our knowledge, this is the first study combining the information on health and nutritional status of 6-59-month-old children in the Philippines collected in two large and representative surveys in 2008. We build a detailed and transparent health economic model describing the lifetime health and cost consequences of iron, vitamin A and zinc deficiencies in these children. Prevalence of MNDs and health and cost consequences are differentiated by 10 SES and 2 age groups. The results are an important contribution to the design, evaluation and choice of the most effective and cost-effective policies aiming at the reduction of MNDs. We carry out a comprehensive sensitivity analysis in order to identify the parameters with the largest effect on the results and to test the overall robustness of the model. The model may in future be adapted to other countries.

Some limitations have to be mentioned. *First*, our approach in identifying the health consequences of MNDs via the results of micronutrient supplementation trials has two potential caveats: The supplementation of vitamin A and zinc in high doses may have a “pharmacological effect” which may lead to an *overestimation* of the adverse health consequences. High doses of vitamin A are for example employed in the treatment of measles, malaria, diarrhea and respiratory diseases and high doses of zinc are employed in the treatment of diarrhea in children. However, some children with severe deficiencies and consequent health impairments are excluded from supplementation trials as it would be unethical to give these children a placebo supplement. This may lead to an *underestimation* of the health consequences of MNDs. These uncertainties are at least in part explored in the probabilistic SA. *Second*, the consequences of some MNDs can only be quantified with single studies and not with systematic reviews. This is particularly the case for the long-term consequences of IDA on cognitive scores and the consequent reduction of adult wages. *Third*, we compute future production losses with an average future wage based on the current average wage and the assumption of a constant productivity growth rate. We thus assume that future wages of children will converge to the average wage. We believe that this assumption can be supported in face of the uncertainty of the long-term development of a rapidly evolving emerging country and the circumstance that lower current wages in lower SES are in part due to the long term consequences of malnutrition in childhood. In the sensitivity analysis we show that dropping this assumption and assuming productivity growth rate adjusted future wages equal to the current SES specific wages would reduce production losses by 37%.

### Comparison with previous research

Our results appear in line with most results of previous studies although a comparison is often difficult because of differences in population, cost-categories, micronutrients and countries considered. A direct comparison can be made with 2 studies which include the calculation of the costs of MNDs in the Philippines: Zimmermann and Qaim [59] examined the DALY losses due to VAD in children below 7 years of age in the Philippines based on NNS data of 1993 and 1998. They calculate a loss of 139,518 DALYs in children due to mortality and disability caused by VAD. This loss is higher than the 57,443 DALYs lost due to VAD in 2008 according to our study. The divergence can, however, at least in part be explained by the difference in the populations (we do not include 0–5 and 60-84-month-old children) and the decrease in the prevalence of VAD in the Philippines over the previous 10–15 years which led to the elimination of some severe health consequences of VAD such as nightblindness, Bitot’s spots and corneal scars. A study by Meenakshi et al. [60] estimates losses of 70,000 DALYs due to VAD and of 80,000 DALYs due to ZnD for Filipino children below the age of 6 years based on surveys carried out in the 1990s and early 2000s.

An apparent difference between our results and those of previous studies is the lower magnitude of DALY losses due to ZnD (7% of total DALY losses in our study) in comparison to those of VAD (47%) and IDA (46%). In the important study by Black et al. [4], distribution of global DALY losses among these MNDs looks, for example, quite different with 40% of losses attributed to ZnD, 55% to VAD and only 5% to IDA. Apart from differences in the populations and countries considered, the higher DALY losses due to IDA in our study are mainly due to the inclusion of DALY losses attributed to impaired cognitive development, while the lower share of losses due to ZnD is mainly due to the absence of an impact of ZnD on mortality. This absence of an impact on mortality is based on the findings of a recent systematic review of zinc supplementation trials [15], which shows no significant effect of zinc on mortality when given in conjunction with other micronutrients.

### Policy implications

Our results show that the costs of MNDs are highly concentrated in the lower SES and the younger age group of children. Costs are 5 times higher in the poorest third of households than in the wealthiest third. The difference in cost per child between the 6-23-month age group and the 24-59-month age group is even larger with a 4.4-fold difference in production losses and a 6.5-fold difference in intangible costs. These results are important because interventions focusing on the SES and the age classes with most severe deficiencies and highest costs are likely to have a higher health impact and to be more cost-effective.

The *invisible* or *hidden* nature of the costs of MNDs is particularly apparent for IDA, as 84% of DALY losses and nearly all production losses occurring in future lifetime are triggered by IDA between 6 and 23 months of age. Without any specific knowledge of the links between nutrition and future cognitive development of their child, the parents will have no reason to improve the diet of the child. The reasons why parents do not invest sufficiently in the future health of their children although the cost of prevention appears small relative to its future benefits have, for example, been investigated in the research on the use of mosquito nets for malaria prevention [61] or vaccinations [62]. Policies aimed at reducing MNDs in children living in poor households should take account of the informational, behavioural and economic obstacles highlighted in this literature.

The much higher prevalence of MNDs in lower SES may be one of the factors leading to a health-based poverty trap [63]. Because of inadequate nutrition, children of the poor are more likely to be ill with diarrhea and respiratory infections. Their families will have to spend more on medical care and may not be able to provide them with adequate care. Due to the impact of MNDs on physical and cognitive development, many of these children will not be able reach their full potential and become poor parents in their later life.

## Conclusions

MNDs lead to substantial costs in 6-59-month-old children in the Philippines. These costs are dominated by future production losses and intangible costs. Costs are highly concentrated in the lower SES and in 6-23-month-old children. These results may have important implications for the design, evaluation and choice of the most effective and cost-effective policies aimed at the reduction of MNDs.

## Abbreviations

DALY: Disability adjusted life year; DHS: Demographic and Health Survey; FNRI: Food and Nutrition Research Institute; IDA: Iron deficiency anemia; MNDs: Micronutrient deficiencies; NNS: National Nutrition Survey; PAF: Population attributable fraction; RR: Relative Risk; SA: Sensitivity analysis; SD: Standard deviation; SES: Socio-economic strata; USD: United States Dollar; VAD: Vitamin A deficiency; ZnD: Zinc deficiency.

## Competing interests

The authors declare that they have no competing interests.

## Authors’ contributions

SW & UB: Designed the study. SW: designed the cost of illness model and prepared the first draft of the manuscript. RP: Was responsible for data analysis, made major contributions to the cost of illness model and provided comments on the manuscript KE: Made major contributions to the health economic model and provided comments on the manuscript. OM: Analyzed literature and participated in the data analysis. MC: Provided important inputs to the cost of illness model and supervised the analysis of nutrition data. IA: Was responsible for the analysis of nutrition data. All authors revised the manuscript critically for important intellectual content and approved the final version.

## Pre-publication history

The pre-publication history for this paper can be accessed here:

http://www.biomedcentral.com/1471-2458/13/1167/prepub

## Supplementary Material

Additional file 1 Details of the economic model. Click here for file
