# Supplementary material for: Burden of micronutrient deficiencies by socio-economic strata in children aged 6 months to 5 years in the Philippines
Source: BMC Public Health. 2013 Dec 11;13:1167. doi: 10.1186/1471-2458-13-1167 (PMC3867423; doi:10.1186/1471-2458-13-1167)
Supplement: Additional file 1 — Details of the economic model. [file 1471-2458-13-1167-S1.pdf]

Appendix 1: Details of the economic model

1 Prevalence of MND

$$p_{ij}^{z} = F_{Norm}(th^{z}, \mu_{ij}^{Se}, \sigma_{ij}^{Se}) \tag{1}$$

With p = prevalence of deficiency z in socioeconomic group i and age group j, $i \in$ {SES1, SES2, ... , SES10} and $j \in$ {6m-11m, 12m-23m, 24-59m}. F_{Norm} = cdf of normal distribution at the threshold for deficiency th given the mean serum level $\mu_{Se_{ij}}$ and standard deviation of serum level $\sigma_{Se_{ij}}$. Prevalence for IDA is adjusted by multiplying p_{ij}^{anemia} with the share of anemia attributable to iron deficiency. The number of deficient children in each group is calculated by multiplying the p_{ij} prevalence rate with the population size of each group (pop_{ij}).

2 Population Attributable Fractions

The share of illness that is caused by a deficiency is determined by the population attributable fraction (PAF). The PAF depends on the prevalence of the deficiency p_{ij}^{z} and the relative risk of the illness (r_k). PAFs were calculated according to equation 2.

$$paf_{ij}^{z,k} = \frac{p_{ij}^{z}(r_k - 1)}{1 + (r_k - 1)p_{ij}^{z}} \tag{2}$$

This is Levin's classical formula for the attributable fraction [31].

3 Number of Deaths

The number of deaths is calculated as follows

$$mort_{ij}^{z} = pop_{ij} * mort_{ij} * paf_{ij}^{z,mort} \tag{3}$$

With $mort$ being the mortality rate.

4 Number of DALYs

4.1 Mortality

In order to calculate the number of DALYs in the first year due to mortality, the time of death within the current year must be assumed. For each age group, time of death is assumed to be in the middle of the considered age period. Thus for 1 year cohorts half a DALY accrues for each death in the first year, while for half year cohorts only a quarter of a DALY accrues.

4.2 Physical Activity

The estimates of the effect of IDA on physical activity was obtained by comparing groups with and without IDA. The assumed effect is thus an average effect of the deficient. Consequently this average effect is applied to all children with IDA. DALYs are calculated as follows (4). For the half year cohort the calculated number of DALYs is then divided by two.

$$DALY_{phys.act.,ij} = n_{ij}^{mod.ane.} * dw_{mod.ane.,ij}^{phys.act.} + n_{ij}^{sev.ane.} * dw_{sev.ane.,ij}^{phys.act.} \tag{4}$$

With $n_{mod.ane.}$ = Number of children with moderate anemia, $n_{sev.ane.}$ = number of children with severe anemia, $dw_{mod.ane.}^{phys.act.}$ = DALY weight of reduced physical activity for the moderately anemic and $dw_{sev.ane.}^{phys.act.}$ = DALY weight of reduced physical activity for the severely anemic.

Impairement of physical activity is assumed to be temporary.

4.3 Mental Development

The calculation of first year DALYs due to impaired mental development follows the same pattern as for physical activity (5).

$$DALY_{ment.dev.} = n_{mod.ane.} * dw_{mod.ane.}^{ment.dev.} + n_{sev.ane.} * dw_{sev.ane.}^{ment.def.} \tag{5}$$

With $dw_{mod.ane.}^{ment.dev.}$ = DALY weight of reduced mental development for the moderately anemic and $dw_{sev.ane.}^{ment.def.}$ = DALY weight of reduced mental development for the severely anemic

Future DALYs accrue until the end of the expected lifetime for a period of $life\ exp. - age$.

4.4 Stunting

From the literature we know the effect of ZnD on the height for age z-score (HAZ). The prevalence of stunting is calculated analogously to the prevalence of MNDs. The share of stunting (definded as HAZ < -2) attributable to ZND is calculated by estimating the mean HAZ score with and without deficiency and comparing prevalence rates of stunting at the two values (6)-(9).

$$v_{ij}^{stunt,znd} = F_{Norm}(th^{stunt}, \mu_{ij}^{def}, \sigma_{ij}^{haz}) - F_{Norm}(th^{stunt}, \mu_{ij}^{ndef}, \sigma_{ij}^{haz}) \tag{6}$$

The different μ_{ij} are calculated as follows (7)

$$HAZ_{ij} = p_{ij}^{znd} \mu_{ij}^{def} + (1 - p_{ij}^{znd}) * (\mu_{ij}^{def} + e_{znd}^{stunt}) \tag{7}$$

This equation can be solved for μ_{ij}^{def} and μ_{ij}^{ndef} (8) & (9)

$$\mu_{ij}^{def} = HAZ_{ij}^{mean} + (p_{ij}^{znd}) * e_{znd}^{stunt} \tag{8}$$

$$\mu_{ij}^{ndef} = \mu_{ij}^{def} + e_{znd}^{stunt} \tag{9}$$

With v^{stunt} = prevalence of stunting, th^{stunt} threshold of HAZ-score for stunting, μ^{def} = mean value of HAZ score for the deficient, μ^{ndef} = mean value of HAZ score for the non-deficient and e_{znd}^{stunt} = effect of ZnD on HAZ-score. DALYs are then calculated as follows (10).

$$DALY_{ij}^{stunt,znd} = pop_{ij} * v_{ij}^{stunt,znd} * dw^{stunt} \tag{10}$$

4.5 Number of Days with Illness

4.5.1 Diarrhea & LRTI

The calculation of DALYs due to illness is based on the number of days with the respective illness (11).

$$ndays_{ij}^{k} = v_{ij}^{k} * paf_{ij}^{z,k} * \frac{365}{14 + dur^{k}} * dur^{k} * pop_{ij} \quad (11)$$

With v^k = prevalence of illness k, dur = duration of illness k. Number of DALYs is then calculated by dividing the number of days by 365 and multiplying by the DALY weight. From DHS data we know the number of illess episodes in the last 14 days. Thus any illness with onset in a period of $14 + dur^k$ days is registered. By dividing with this term and multiplying by 365 the number of yearly episodes is calculated. In order for this calculation to be valid, we must assume that disease prevalence and duration is constant over any two consecutive weeks in the year. We also assume that any child is sick at most once in the observed two weeks period. DALYs are calculated as follows.

$$DALY_{ij}^{k} = \frac{ndays_{ij}^{k}}{365} * dw^{k} \quad (12)$$

with dw^k = disability weight of illness k.

4.5.2 Measles

For measles no group specific prevalence rates are available. These are estimated using the prevalence for the general population and the group specific mealses vaccination rates.

$$v_{j}^{mea} = \frac{v^{mea}}{100'000} * (\sum_{ij} pop_{ij}) * \frac{s_j}{\sum_i pop_{ij}} \quad (13)$$

With v_j^{mea} = measles prevalence s_j = share of population in age group j. Based on these age specific prevalence rates, the group specific prevalence is calculated by weighting the prevalence rate with the measles vaccination rate.

$$v_{ij}^{mea} = \frac{v_{j}^{mea}}{\frac{pop_{ij}}{\sum_i pop_{ij}}} * \frac{(1 + vac_{ij} * (ivac - 1) * \frac{pop_{ij}}{\sum_i pop_{ij}}}{\sum_i (1 + vac_{ij} * (ivac - 1) * \frac{pop_{ij}}{\sum_i pop_{ij}}} \quad (14)$$

This simplifies to

$$v_{ij}^{mea} = v_{j}^{mea} * \frac{(1 + vac_{ij} * (ivac - 1)}{\sum_i (1 + vac_{ij} * (ivac - 1) * \frac{pop_{ij}}{\sum_i pop_{ij}}} \quad (15)$$

Days of illness and DALYs are then calculated as follows

$$ndays_{ij}^{mea} = pop_{ij} * v_{ij}^{mea} * paf_{ij}^{vad,mea} * dur^{mea} \quad (16)$$

$$DALY_{ij}^{mea} = \frac{ndays_{ij}^{mea}}{365} * dw^{mea} \quad (17)$$

With dw^{mea} = disability weight for measles, $ndays^{mea}$ = number of days with measles, dur^{mea} = average duration of a measles episode. Given that patients are immune to future measles infections after illness, we assume that the number of measles cases corresponds to the number of infections of new patients.

5 Income Loss

5.1 Current

Calculation of current yearly income losses is implemented as described in formula (18).

$$incl_{ij}^{z,k} = pop_{ij} * v_{ij}^{z} * paf_{ij}^{z,k} * dur_{care}^{k} * wage_i * \frac{5}{7} * (w_{part} - 0.5) * 2 \quad (18)$$

With $wage_i$ = Daily wage in dollars in SES_i, dur_{care}^{k} = number of days off work to care for a child with illness k. w_{part} = share of work force participation. The term $\frac{5}{7}*(w_{part}-0.5)*2$ adjusts the wage rate for the probability that a day of illness falls on a weekend and for the share of work force participation. We assume that only in $(w_{part} - 0.5) * 2$ share of all households are both parents working. This depends on the assumption that all men are employed.

5.2 Future

Calculations of future income loss are based on 240.7143 work days per year. Children are assuemed to start working when they turn 15 and leave paid employment at age 65. Future income is expected to grow at a rate of 1.66% per year, which corresponds to the real growth of per capita GDP over the past 10 years. The level of work force participation is assumed to be constant.

Future income is affected by stunting due to ZnD and impaired mental development due to IDA. Effect of stunting on wage is obtained from literature. Mortality also leads to losses in future production.

For impared mental development due to IDA, the effect on IQ is known from literature as well as the effect from a lower IQ on wage. In a first step the number of standard deviations of IQ lost due to IDA are calculated. This in turn is multiplied by the effect of an IQ that is lower by 1 SD on the wage and the yearly wage rate.

6 Treatment Costs

For all illnesses mean treatment costs per episode are estimated based on DHS data. Treatment costs are thus calculated by multiplying the number of episodes with the average costs.

7 Discounting

Future losses are discounted using the formula for geometric rows (19).

$$a_0 \sum_{k=0}^{n} q^k = a_0 \frac{q^{n+1} - 1}{q - 1} \tag{19}$$

For the model a function has been defined that calculates the n-th partial sum of a geometric progression and subtracts the (b-1)-th partial sum. This allows for losses that only accrue from time b to time n. The formula looks as follows (20)

$$a_0 \sum_{k=b}^{n} q^k = a_0 \frac{q^{n+1} - q^b}{q - 1} \tag{20}$$

with a_0 = future loss, $q = \frac{1+g}{1+r}$, g = growth rate of loss, r = discount rate, b = first period of loss, n = last period of loss.
